# Assessing the Reliability of Hysteroscopic Sampling Methods for Diagnosing Atypical Endometrial Hyperplasia †

**DOI:** 10.3390/cancers17183036

**Published:** 2025-09-17

**Authors:** Luca Giannella, Francesco Piva, Giovanni Delli Carpini, Jacopo Di Giuseppe, Matteo Giulietti, Erica Dugo, Francesco Sopracordevole, Anna Del Fabro, Nicolò Clemente, Barbara Gardella, Giorgio Bogani, Orsola Brasile, Ruby Martinello, Marta Caretto, Alessandro Ghelardi, Gianluca Albanesi, Guido Stevenazzi, Paolo Venturini, Maria Papiccio, Marco Cannì, Maggiorino Barbero, Massimiliano Fambrini, Veronica Maggi, Stefano Uccella, Arsenio Spinillo, Francesco Raspagliesi, Pantaleo Greco, Tommaso Simoncini, Felice Petraglia, Andrea Ciavattini

**Affiliations:** 1 Gynecologic Section, Woman’s Health Sciences Department, Polytechnic University of Marche, 60123 Ancona, Italy; luca.giannella@ospedaliriuniti.marche.it (L.G.); giovanni.dellicarpini@ospedaliriuniti.marche.it (G.D.C.); jacopo.digiuseppe@ospedaliriuniti.marche.it (J.D.G.); 2 Department of Specialistic Clinical and Odontostomatological Sciences, Polytechnic University of Marche, 60131 Ancona, Italy; f.piva@univpm.it (F.P.); m.giulietti@univpm.it (M.G.); e.dugo@pm.univpm.it (E.D.); 3 Gynecologic Oncology Unit, IRCCS—Centro di Riferimento Oncologico di Aviano, 33081 Aviano, Italy; fsopracordevole@cro.it (F.S.); anna.delfabro@cro.it (A.D.F.); nicolo.clemente@cro.it (N.C.); 4 Department of Obstetrics and Gynecology, Fondazione IRCCS Policlinico San Matteo, Università Degli Studi di Pavia, 27100 Pavia, Italy; barbara.gardella@gmail.com (B.G.); spinillo@smatteo.pv.it (A.S.); 5 Gynecological Oncology Unit, Fondazione IRCCS—Istituto Nazionale Tumori, 20133 Milano, Italy; giorgio.bogani@istitutotumori.mi.it (G.B.); raspagliesi@istitutotumori.mi.it (F.R.); 6 Section of Obstetrics and Gynecology, Department of Medical Sciences, University of Ferrara, 44124 Ferrara, Italy; brsrsl@unife.it (O.B.); mrtrby@unife.it (R.M.); grcptl@unife.it (P.G.); 7 Division of Obstetrics and Gynecology, Department of Clinical and Experimental Medicine, University of Pisa, 56124 Pisa, Italy; marta.caretto@phd.unipi.it (M.C.); tommaso.simoncini@med.unipi.it (T.S.); 8 UOC Ostetricia e Ginecologia, Ospedale Apuane, Azienda Usl Toscana Nord-Ovest, 54100 Massa, Italy; alessandro.ghelardi@uslnordovest.toscana.it (A.G.); gianluca.albanesi@uslnordovest.toscana.it (G.A.); 9 Department of Obstetrics and Gynaecology, ASST Ovest MI, Legnano (Milan) Hospital, 20025 Legnano, Italy; guido.stevenazzi@asst-ovestmi.it; 10 Division of Obstetrics and Gynecology, AUSL di Modena, 41012 Carpi, Italy; p.venturini@ausl.mo.it (P.V.); m.papiccio@ausl.mo.it (M.P.); 11 Department of Obstetrics and Gynecology, Asti Community Hospital, 14100 Asti, Italy; mcanni@asl.at.it (M.C.); barberom@tin.it (M.B.); 12 Obstetrics and Gynecology, Department of Experimental, Clinical, and Biomedical Sciences, Careggi University Hospital, University of Florence, 50121 Florence, Italy; massimiliano.fambrini@unifi.it (M.F.); felice.petraglia@unifi.it (F.P.); 13 Department of Obstetrics and Gynecology, University of Verona, 37129 Verona, Italy; veronica.maggi@studenti.univr.it (V.M.); stefano.uccella@univr.it (S.U.)

**Keywords:** atypical endometrial hyperplasia, endometrial cancer, endometrial sampling, hysteroscopically guided biopsy, hysteroscopic endometrial resection

## Abstract

**Background/Objectives**: The diagnosis of atypical endometrial hyperplasia (AEH) is associated with a high rate of concurrent endometrial cancer (EC). This occurrence can be particularly challenging in premenopausal women wishing to become pregnant, as they may be subjected to conservative treatment. The type of endometrial sampling may affect this outcome. Currently, the recommended type of endometrial sampling is under hysteroscopic guidance. There is scant literature regarding the reliability of hysteroscopically guided biopsy (HSC-bio) and hysteroscopic endometrial resection (HSC-res) on this topic. We aimed to assess the underestimation rate of EC in AEH, according to different hysteroscopic sampling methods. The secondary outcome was to evaluate the procedure performance in pre- and postmenopausal women. **Methods**: We conducted a multi-institutional retrospective study that included 536 women diagnosed with AEH who underwent hysterectomy between 2015 and 2020. Patients were divided into two groups based on the initial diagnostic approach for AEH: HSC-bio and HSC-res. The comparison was performed using univariate and multivariate analyses. **Results**: 160/536 women (29.9%) showed EC at hysterectomy. Overall, the following rate of EC underestimation was found: HSC-bio = 32.1%, HSC-res = 24.2%, *p* = 0.07. After adjusting for baseline characteristics using logistic regression analysis, overall, there was no significant association of EC underestimation according to the type of sampling procedure. Interestingly, in premenopausal women, including 161 cases, the rate of EC underestimation in HSC-bio and HSC-res was 28.8% vs. 14.0%, respectively (*p* = 0.034). **Conclusions**: There were no significant differences in EC underestimation between the two hysteroscopic procedures in the entire cohort of women with AEH. Limited to the secondary objective, the significant findings in premenopausal women may be of particular clinical interest, as this population may undergo conservative treatment.

## Simple Summary

The diagnosis of atypical endometrial hyperplasia often coexists with endometrial cancer. This clinical condition can be particularly challenging to manage in premenopausal women seeking pregnancy and eligible for conservative treatment. There is limited literature concerning the reliability of hysteroscopic sampling methods and the potential underestimation of endometrial cancer rates in women diagnosed with atypical endometrial hyperplasia before surgery. Even less is known about the effectiveness of hysteroscopic endometrial sampling according to premenopausal and postmenopausal status, considering confounding factors related to endometrial pathology on this topic. The present study aimed to fill this knowledge gap and revealed that resectoscopic endometrial biopsy has the lowest rate of underestimating endometrial cancer in premenopausal women.

## 1. Introduction

Atypical endometrial hyperplasia (AEH) is a challenging condition for clinicians. This diagnosis may include concurrent endometrial cancer (EC) in up to 40% of cases [1,2,3]. Although the majority of cases are stage 1 invasive cancer, approximately 10% may present with high-risk EC requiring further surgical procedures [3,4,5]. Furthermore, while surgical treatment is the recommended option for postmenopausal women, conservative treatment may be adopted for premenopausal women still seeking pregnancy [6,7,8]. In this latter population, it is therefore even more important to have an accurate diagnosis.

Additionally, EC includes a diagnostic workup, including specific imaging, which is not mandatory for AEH (e.g., magnetic resonance imaging) [9]. Understanding these differences can be crucial in managing expectations and planning for the future.

In AEH, the type of endometrial sampling can affect the correct preoperative diagnosis. In the past, endometrial biopsies were performed with blind sampling, which proved unreliable, sampling on average only half the endometrial cavity and therefore often missing focal lesions [10,11]. Currently, major scientific societies (ACOG, ESGO) recommend performing visualized targeted biopsies to exclude EC in women diagnosed with AEH [7,12].

The two primary endoscopic methods for endometrial sampling are hysteroscopically guided biopsy (HSC-bio) and hysteroscopic endometrial resection (HSC-res). Currently, the most widely used is HSC-bio [7]. However, evidence in the literature has shown that HSC-res is more reliable in ruling out the presence of concomitant EC, since HSC-bio has been shown to have a worse performance in excluding endometrial hyperplasia [13,14,15,16]. There is conflicting evidence in the literature regarding the reliability of these two procedures in diagnosing AEH correctly. These data may be affected by the small sample size of previous studies, especially among women undergoing HSC-res, and by certain confounding risk factors, e.g., age, pre- or menopausal status, body mass index (BMI), diabetes, etc., which are not considered, and that could affect the diagnostic performance of the procedure [13,14]. Currently, there is limited literature concerning the reliability of these two hysteroscopic sampling procedures on this topic, particularly regarding any differences based on pre- or postmenopausal status.

The present study aimed primarily to assess the underestimation rate of EC in AEH, using different hysteroscopic sampling methods (HSC-bio vs. HSC-res), including a comparison of confounding variables. Secondly, the focus was on potential differences in procedure performance between premenopausal and postmenopausal women, as treatment options may vary in clinical practice.

## 2. Materials and Methods

### 2.1. Study Design and Setting

This multi-institutional retrospective study involved thirteen oncology referral centers in Italy. Women diagnosed with AEH after undergoing either office hysteroscopy (HSC-bio) or resectoscopic endometrial biopsy (HSC-res) received definitive surgical treatment within forty days of the first diagnosis, with hysterectomy being the oncological reference standard. The histological assessment of AEH was based on the WHO 2014 classification and includes a dichotomous categorization: atypical and non-atypical endometrial hyperplasia [17,18]. The study period spanned from January 2015 to December 2020. It is essential to note that this study is a secondary analysis of a previous research project that followed the same inclusion and exclusion criteria [19]. An ethics committee approved the original study protocol, so the current secondary analysis did not require additional institutional review board approval [19].

### 2.2. Study Groups and Variables

The sample of women included in the study was divided into two groups: those undergoing HSC-bio and those undergoing HSC-res, which represented the dependent variable. The main indications for performing hysteroscopic examinations in the study participants were abnormal uterine bleeding or endometrial abnormalities identified via ultrasound. Women who underwent HSC-res did so primarily due to the failure of the office hysteroscopy or inadequate tissue sampling during the HSC-bio procedure or due to the presence of contraindications to performing the diagnostic assessment in an outpatient setting.

The following independent variables were considered: age, pre- or postmenopausal status, parity, smoking status, body mass index (BMI), comorbidities (including diabetes, hypertension, or both), presence of Lynch syndrome, history of breast cancer, use of tamoxifen, use of hormone therapy, abnormal uterine bleeding, and ultimately EC stage based on the EC classification system [9]. Following the initial analysis of the independent variables between the two groups, particular attention was given to examining the performance of the procedures in both premenopausal and postmenopausal women. Descriptive data concerning the stage of endometrial carcinoma cases, categorized by endometrial sampling method and menopausal status (pre- or postmenopausal), were provided.

### 2.3. Procedure Details

All office hysteroscopies (HSC-bio) were performed in an outpatient setting without anesthesia and under vaginoscopy, utilizing a 5 mm continuous-flow sheath with a 30° viewing angle, and using saline solution as a distension medium. Endometrial sampling was performed using 5 French semi-rigid forceps inserted through the working channel of the hysteroscope (Bettocchi Hysteroscope, Karl Storz^®^, Tuttlingen, Germany).

In contrast, resectoscopic endometrial biopsies (HSC-res) were conducted in a hospital setting. The procedure was performed in the operating room under general anesthesia, employing a 9 mm continuous-flow sheath with a 12° viewing angle. Initially, the cervical canal was dilated to 9.5 mm, and then the instrument was introduced, and a bipolar operating loop of 26 French was used for biopsy sampling of the endometrium only, and saline solution was used as as a distension medium (Karl Storz^®^, Tuttlingen, Germany).

### 2.4. Sample Size Calculation

The sample size calculation was performed to compare two proportions based on the primary outcome: the rate of concurrent EC in women with AEH, utilizing two different endometrial sampling methods: HSC-bio and HSC-res. According to the existing literature on this subject [13,14,19], we anticipated an average missed EC rate of approximately 36% for women undergoing HSC-bio and 23% for those using HSC-res, with a ratio of 2:1, as HSC-bio is the more commonly used method. With a Type I error (Alpha, significance) set at 0.05 and a Type II error (Beta, 1-Power) at 0.20, the minimum required sample size for the study is 440 cases, divided between the two methods (293 HSC-bio and 147 HSC-res) [20].

### 2.5. Statistical Analysis

Continuous independent variables were assessed for distribution using the Kolmogorov–Smirnov test. Variables that did not exhibit a normal distribution were analyzed using the nonparametric Mann–Whitney U test and were presented as medians along with interquartile ranges. Categorical variables were compared using the Chi-squared test and reported as counts and percentages. Significant differences identified in univariate analyses were further examined in a multivariate logistic regression model, which was used to identify variables significantly associated with the endometrial sampling methods employed. In multivariate analysis, we included explanatory variables that showed a *p*-value ≤ 0.1 in the univariate model [21]. Finally, a sub-analysis was performed to compare the rate of EC underestimation between different hysteroscopic techniques in pre- and postmenopausal women.

The MedCalc Statistical Software was used to perform statistical analyses [MedCalc^®^ Statistical Software version 20.305 (MedCalc Software Ltd., Ostend, Belgium; https://www.medcalc.org; 2023)]. A value of *p* < 0.05 was considered statistically significant.

## 3. Results

**Figure 1:**
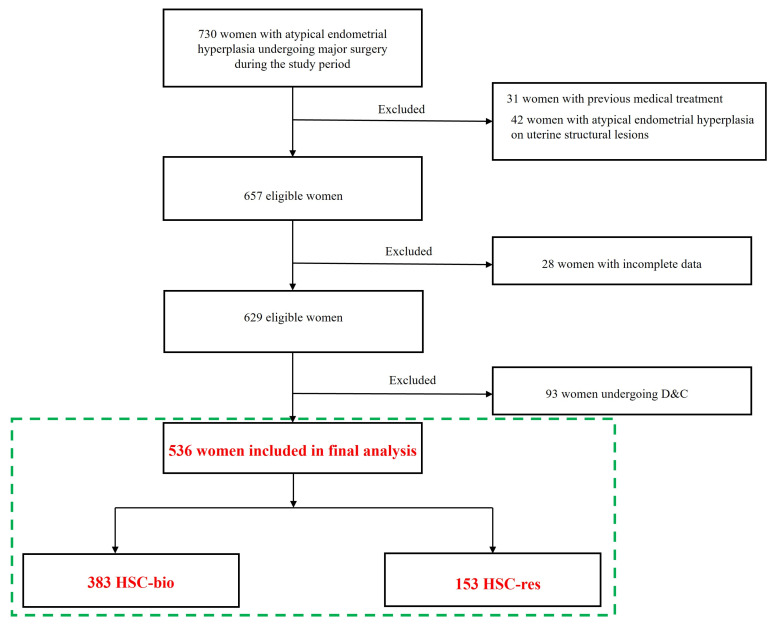
Study Flow-chart.

The present study included 536 women with a preoperative diagnosis of AEH who subsequently underwent definitive treatment. Of these, 383 underwent HSC-bio and 153 HSC-res. Figure 1 shows the study flowchart.

The characteristics of the patients are presented in Table 1. The average age of the participants was 57 years. Among the women studied, 70% were postmenopausal, while 161 patients were premenopausal. Of the premenopausal group, 12 patients (7.5%) were under 40 years of age, 21 patients (13.0%) were under 45 years of age, and 128 patients (79.5%) were 45 years or older. Approximately 70% of the women were classified as overweight or obese, and around 68% experienced abnormal uterine bleeding. Additionally, 383 women (71.5%) underwent HSC-bio procedures, while 153 had HSC-res procedures. The final histological examination revealed an EC rate of 29.9%.

Univariate analysis comparing the independent variables revealed significant differences between the two groups in terms of age and pre- and postmenopausal status (see Table 2). The incidence of endometrial cancer (EC) was lower in the HSC-res group (24.2%) compared to the HSC-bio group (32.1%). However, this finding did not reach statistical significance (*p* = 0.07) (refer to Table 2). No significant differences were observed among the other independent variables analyzed.

To evaluate a possible association of missed ECs with the two hysteroscopic procedures analyzed and adjusted for confounding variables as described in the methods, a multivariate analysis was performed in which the dependent variable was categorized as follows: EC = 1, No-EC = 0 (Table 3). After adjusting for confounders (including age and pre-/postmenopausal status according to univariate analysis, and adding endometrial sampling methods), overall, no significant association was found between EC at final histology and endometrial sampling procedures (Table 3).

Interestingly, when analyzing premenopausal and postmenopausal women separately, a significant difference was observed in the rate of underestimation of EC among premenopausal women. Specifically, in this group, the rate of cancer events for those in the HSC-res group was 14%, compared to 28.8% for women in the HSC-bio group (*p* = 0.034) (see Table 4). No significant differences were found in postmenopausal women (refer to Table 4). There were no significant differences between the independent variables considered in the two groups of endometrial sampling methods for both premenopausal and postmenopausal women (Tables S1 and S2).

The analysis of the final stage of EC cases revealed no significant differences between the two endoscopic procedures (Table 5). Although the comparison did not provide a significant result, Stage I intermediate/high-risk and Stage II EC were missed more frequently in premenopausal women undergoing HSC-bio compared to HSC-res: 6.7% vs. 3.5% (Table 5). Overall, 9 out of 161 premenopausal patients (5.6%) had a missed intermediate- or high-risk EC diagnosis (Table 5). Likewise, in postmenopausal women, there was no significant difference between the final stage of EC and the two hysteroscopic procedures (Table 6). In this last group of patients, the rate of Stage I intermediate/high-risk and Stage II EC was similar in the two groups: 8.9% in the HSC-bio group and 8.3% in the HSC-res group (Table 6).

## 4. Discussion

The present study demonstrated that there was no significant difference between HSC-res and HSC-bio about the rate of EC underestimation in the entire cohort of women with AHE. Interestingly, the secondary objective of the study showed a significantly lower rate of EC in premenopausal women with AEH undergoing HSC-res compared to HSC-bio.

The diagnosis of AEH raises concerns about the possible concurrent presence of endometrial cancer. Our observed rate of approximately 30% is slightly lower than recent data from a systematic review and meta-analysis [22]. The use of only hysteroscopic methods could likely explain this slight difference, since we did not use blind procedures, which are less accurate [10,11]. While the diagnosis of EC in postmenopausal women at final histological assessment does not present a significant issue—given that treatment is typically surgical and can include the use of sentinel lymph node biopsy—it poses a more complex clinical challenge for premenopausal women who wish to conceive [4,5,7,23]. In such cases, an accurate diagnosis is crucial.

Currently, an endometrial biopsy in these cases should be performed under hysteroscopic guidance [7]. The literature on this subject presents inconclusive results, often derived from small sample sizes subjected to endoscopic biopsy without adequately accounting for confounding variables related to endometrial pathology [13,14,24]. A previous study included 208 women with AEH and compared the rate of concurrent EC in patients undergoing D&C (75 cases), HSC-bio (75 cases), and HSC-res (58 cases). In that study, HSC-res showed the lowest rate of EC. However, the cases subjected to endoscopic sampling were not so large [14]. Furthermore, in a systematic review and meta-analysis, Bourdel et al. evaluated over 1000 women with AEH who underwent various types of endometrial sampling [13]. They aimed to identify the method with the lowest rate of EC underestimation. The results indicated that hysteroscopic resection (HSC-res) was the most accurate method when compared to hysteroscopic biopsy (HSC-bio) and dilation and curettage (D&C). They showed a rate of EC underestimation in HSC-bio, HSC-res, and D&C of 45.3%, 5.8%, and 32.7%, respectively [13]. However, out of the more than 1000 cases analyzed, only 99 cases of HSC-bio and 23 instances of HSC-res were included in the analysis [14]. Hence, the idea of performing a more comprehensive comparison using a larger sample size and ensuring that the samples accounted for confounding variables related to endometrial pathology.

Based on our analysis, the majority of EC cases were categorized as low-risk. It has been established that both AEH and low-risk EC can be managed conservatively [7,12]. However, it is essential to note that the rates of complete recovery and recurrence differ between AEH and invasive cancer, the latter being higher for EC [25]. For example, the disease recurrence rates are approximately 41% for grade 1 EC and 26% for AEH [26]. Therefore, conservative treatment does not have the same impact in both conditions, and this should be taken into account by clinicians managing these patients. Finally, these different outcomes should be communicated to women through careful counseling to discuss the best treatment option with each patient.

Moreover, our results indicate that 5.6% of premenopausal women with concurrent EC had intermediate- or high-risk tumors, and most of them were in the group undergoing HSC-bio. This information raises questions about the appropriate management of this population. Overall, our sample included 161 premenopausal patients. Of these, approximately 21% were under 45, which nowadays represents a life period to desire pregnancy. Additionally, it is worth noting that the average age of childbirth has significantly increased over the years, with many women giving birth even after 45 [27,28]. Given these data, it could be argued that the best approach for premenopausal women undergoing conservative treatment is to obtain a diagnosis of AEH through endometrial sampling performed with HSC-res, even after a diagnosis based on HSC-bio. The rationale behind this is that the outpatient procedure typically yields a smaller amount of tissue than HSC-res, making it less representative of the endometrial lesion. So, HSC-res may be indicated to provide a more reliable histological reference standard in women with AEH who are eligible for conservative treatment.

However, in this scenario, it is essential to clarify the acceptability, safety, and risk of complications associated with endometrial resection in women seeking pregnancy. In this context, it is crucial to emphasize that a diagnostic resectoscopic endometrial biopsy does not impact fertility [6,29]. The risk of intrauterine adhesions is very low after diagnostic sampling [30,31]. Furthermore, it has been reported that resectoscopic operative hysteroscopy can be used as a fertility-sparing treatment method in women seeking pregnancy, in conjunction with progestin-based medical therapy, for both AEH and EC [32]. Finally, if adhesions do occur and impair fertility, hysteroscopic adhesiolysis treatment restores regular menstrual flow, making subsequent pregnancies possible in most cases [30,33].

Conversely, it is unclear why HSC-res does not perform as well in postmenopausal women compared to the outpatient HSC-bio method. One possible explanation is that postmenopausal women have less tissue available for sampling, regardless of the technique used. As a result, the difference in the amount of tissue obtained by the two methods may be less pronounced than in premenopausal women, partly explaining the slight difference in reliability. It is important to emphasize that further investigations are required to clarify this unclear difference.

Another aspect worth noting is that HSC-res requires anesthesia and hospitalization and often involves longer waiting times compared to HSC-bio. Thus, each option should include a careful assessment of costs, risks, and benefits. In this context, outpatient procedures may be adopted that utilize miniaturized hysteroscopes with operating loops. Although these tools may not be available in all clinical divisions, they should be implemented because they can facilitate more tissue sampling without the need for anesthesia, allowing for immediate execution in an outpatient environment [34]. Furthermore, possible associations between the molecular characterization of the missed EC and the sampling method used could represent another interesting aspect. This data, if it showed significant associations, could be of considerable clinical use for choosing the most appropriate type of endometrial sampling.

The present study has several limitations: (i) its retrospective nature; (ii) outpatient and inpatient procedures have not been standardized a priori; (iii) it is unknown whether only selected areas were biopsied or whether and when random biopsies were also performed; (iv) the total amount of tissue sampled may differ (e.g., in pre- or postmenopausal women); (v) based on the study design (including only women with a pre-operative diagnosis of AEH), the exact diagnostic accuracy of the two hysteroscopic methods could not be provided. Strengths include the large sample analyzed, which consists of only hysteroscopic biopsies. The focus on pre- or postmenopausal status is novel in this field. Furthermore, the histological reference standard representative of the final endometrial lesion should be emphasized, given that the final diagnosis was made on hysterectomy specimens.

## 5. Conclusions

Overall, the primary objective of the present study showed no significant difference between HSC-res and HSC-bio in the rate of EC underestimation in women with AEH. However, the significant findings in premenopausal women are particularly engaging, as this population may undergo conservative treatment: in premenopausal women wishing to conceive, HSC-res may be indicated to provide a more reliable histological reference standard. This data needs to be confirmed by a prospective study. To compare further endometrial sampling methods, miniaturized hysteroscopes with operating loops could be added to office sampling methods.

## Figures and Tables

**Table 1:** Patient characteristics.

Independent Variables	*n* (%) (Sample Size = 536)
Age (median and interquartile ranges)	57.0 (51.0–66.0)
Postmenopause	375 (70.0)
Premenopause	161 (30.0)
Nulligravid	104 (19.4)
Smoking habit	107 (20)
Body Mass Index	
<19	12 (2.2)
19.0–24.99	143 (26.7)
25.0–29.99	171 (31.9)
≥30	210 (39.2)
Comorbidity	
Diabetes	14 (2.6)
Hypertension	176 (32.8)
Diabetes + Hypertension	40 (7.5)
Lynch Syndrome	8 (1.5)
Previous breast cancer	81 (15.1)
Tamoxifen users	31 (5.8)
Hormonal therapy	
OC	27 (5.0)
HRT	16 (3.0)
Indication for hysteroscopy	
Abnormal uterine bleeding Endometrial abnormalities at ultrasound	363 (67.7) 173 (32.3)
Endometrial sampling methods	
HSC-bio	383 (71.5)
HSC-res	153 (28.5)
Endometrial Cancer	160 (29.9)

HSC-bio: hysteroscopically guided biopsy; HSC-res: hysteroscopic endometrial resection; OC: oral contraceptive; HRT: hormonal replacement therapy.

**Table 2:** Distribution of independent variables according to different hysteroscopic endometrial sampling methods.

Univariate Analysis			
Independent Variables	HSC-bio (383) *n* (%)	HSC-res (153) *n* (%)	*p*-Value
Age (median and interquartile ranges)	57 (52.0–68.0)	55 (50.0–63.0)	0.005
Menopausaul status			0.021
Postmenopause	279 (72.8)	96 (62.7)	
Premenopause	104 (27.2)	57 (37.3)	
Nulligravid	73 (19.1)	31 (20.3)	0.751
Smoking habit	74 (19.3)	33 (21.6)	0.557
Body Mass Index (median and interquartile ranges)			0.143
<19.0	5 (1.3)	7 (4.6)	
19.0–24.99	104 (27.2)	39 (25.5)	
25.0–29.99	122 (31.9)	49 (32.0)	
≥30	152 (39.7)	58 (37.9)	
Comorbidity			0.162
Diabetes	11 (2.9)	3 (2.0)	
Hypertension	134 (35)	42 (27.5)	
Diabetes + Hypertension	31 (8.1)	9 (5.9)	
Lynch Syndromes	6 (1.6)	2 (1.3)	0.823
Previous breast cancer	55 (14.4)	26 (17.0)	0.442
Tamoxifen users	20 (5.2)	11 (7.2)	0.378
Hormonal therapy users			0.121
OC	14 (3.7)	12 (7.8)	
HRT	12 (3.1)	4 (2.6)	
Indication for hysteroscopy			0.937
Endometrial abnormalities at ultrasound	124 (32.4)	49 (32.0)	
Abnormal uterine bleeding	259 (67.6)	104 (68.0)	
EC at hysterectomy	123 (32.1)	37 (24.2)	0.070

EC: endometrial cancer; HRT: hormonal replacement therapy. HSC-bio: hysteroscopically guided biopsy; HSC-res: hysteroscopic endometrial resection; OC: oral contraceptive.

**Table 3:** Multivariate analysis assessing associations between independent variables and endometrial cancer.

Variables	Odds Ratio	Confidence Interval	*p*-Value
Age	1.02	0.99–1.04	0.051
Postmenopause	Ref.	-	-
Premenopause	0.90	0.53–1.54	0.712
HSC-bio	Ref.	-	-
HSC-res	0.72	0.47–1.11	0.144

HSC-bio: hysteroscopically guided biopsy; HSC-res: hysteroscopic endometrial resection.

**Table 4:** Endometrial cancer rate in pre- and postmenopausal women according to different endometrial sampling methods.

Variable	HSC-bio (104) *n* (%)	HSC-res (57) *n* (%)	*p*-Value
Endometrial cancer cases (Premenopausal women)	30 (28.8)	8 (14.0)	0.034
**Variable**	**HSC-bio** **(279)** ***n* (%)**	**HSC-res** **(96)** ***n* (%)**	***p*-Value**
Endometrial cancer cases (Postmenopausal women)	93 (33.3)	29 (30.2)	0.573

HSC-bio: hysteroscopically guided biopsy; HSC-res: hysteroscopic endometrial resection.

**Table 5:** Final stage of endometrial cancer according to different endometrial sampling methods in premenopausal women.

Variable	Endometrial Sampling Methods		
Final Stage	HSC-bio (104) *n* (%)	HSC-res (57) *n* (%)	*p*-Value
			0.168
Stage I intermediate/high-risk	5 (4.8)	2 (3.5)	
Stage II	2 (1.9)	0 (0.0)	
Stage I low-risk	23 (22.1)	6 (10.5)	
No cancer	74 (71.2)	49 (86.0)	

HSC-bio: hysteroscopically guided biopsy; HSC-res: hysteroscopic endometrial resection.

**Table 6:** Final stage of endometrial cancer according to different endometrial sampling methods in postmenopausal women.

Variable	Endometrial Sampling Methods		
Final Stage	HSC-bio (279) *n* (%)	HSC-res (96) *n* (%)	*p*-Value
			0.926
Stage I intermediate/high-risk	23 (8.2)	7 (7.3)	
Stage II	2 (0.7)	1 (1.0)	
Stage I low-risk	68 (24.4)	21 (21.9)	
No cancer	186 (66.7)	67 (69.8)	

HSC-bio: hysteroscopically guided biopsy; HSC-res: hysteroscopic endometrial resection.

## Data Availability

The data presented in this study are available on request from the corresponding author.

## Supplementary Materials

The following supporting information can be downloaded at https://www.mdpi.com/article/10.3390/cancers17183036/s1, Table S1: Distribution of independent variables according to different hysteroscopic endometrial sampling methods in premenopausal women; Table S2: Distribution of independent variables according to different hysteroscopic endometrial sampling methods in postmenopausal women.
