# Supplementary material for: Assessing the Reliability of Hysteroscopic Sampling Methods for Diagnosing Atypical Endometrial Hyperplasia
Source: Cancers (Basel). 2025 Sep 17;17(18):3036. doi: 10.3390/cancers17183036 (PMC12468639; doi:10.3390/cancers17183036)
Supplement: Supplementary file 1 [file cancers-17-03036-s001.zip › cancers-3849237-supplementary.pdf]

Table S1. Distribution of independent variables according to different hysteroscopic endometrial sampling methods in premenopausal women.

**Independent Variables**	**HSC-bio (104) n (%)**	**HSC-res (57) n (%)**	***P value***
**Age (median and interquartile ranges)**	49.5 (47.0-52.0)	48.0 (45.0-51.0)	*0.162*
**Nulligravid**	34 (32.7)	12 (21.1)	*0.119*
**Smoking habit**	22 (21.2)	10 (17.5)	*0.584*
**Body Mass Index (median and interquartile ranges)**			*0.113*
< 19.0	1 (1.0)	4 (7.0)	
19.0-24.99	38 (36.5)	20 (35.1)	
25.0-29.99	37 (35.6)	23 (40.4)	
≥ 30	28 (26.9)	10 (17.5)	
**Comorbidity**			*0.468*
Diabetes	3 (2.9)	0 (0.0)	
Hypertension	18 (17.3)	7 (12.3)	
Diabetes + Hypertension	3 (2.9)	2 (3.5)	
**Lynch Syndromes**	5 (4.8)	1 (1.8)	*0.329*
**Previous breast cancer**	6 (5.8)	7 (12.3)	*0.148*
**Tamoxifen users**	3 (2.9)	4 (7.0)	*0.220*
**Hormonal therapy users**			*0.364*
OC	9 (8.7)	6 (10.5)	
HRT	0 (3.1)	1 (1.8)	
**Indication for hysteroscopy**			0.410
Endometrial abnormalities at ultrasound	22 (21.2)	9 (15.8)	
Abnormal uterine bleeding	82 (78.8)	48 (84.2)	

HRT: hormonal replacement therapy. HSC-bio: hysteroscopically guided biopsy; HSC-res: hysteroscopic endometrial resection; OC: oral contraceptive

Table S2. Distribution of independent variables according to different hysteroscopic endometrial sampling methods in postmenopausal women.

**Independent Variables**	**HSC-bio (279) n (%)**	**HSC-res (96) n (%)**	***P value***
**Age (median and interquartile ranges)**	49.5 (47.0-52.0)	48.0 (45.0-51.0)	*0.162*
**Nulligravid**	39 (14.0)	19 (19.8)	*0.174*
**Smoking habit**	52 (18.6)	23 (24.0)	*0.261*
**Body Mass Index (median and interquartile ranges)**			*0.508*
< 19.0	4 (1.4)	3 (3.1)	
19.0-24.99	66 (23.7)	19 (19.8)	
25.0-29.99	85 (30.5)	26 (27.1)	
≥ 30	124 (44.4)	48 (50)	
**Comorbidity**			*0.587*
Diabetes	8 (2.9)	3 (3.1)	
Hypertension	116 (41.6)	35 (36.5)	
Diabetes + Hypertension	28 (10.0)	7 (7.3)	
**Lynch Syndromes**	1 (0.4)	1 (1.0)	*0.428*
**Previous breast cancer**	49 (17.6)	19 (19.8)	*0.625*
**Tamoxifen users**	17 (6.1)	7 (7.3)	*0.220*
**Hormonal therapy users**			*0.131*
OC	6 (2.2)	6 (6.2)	
HRT	12 (4.3)	3 (3.1)	
**Indication for hysteroscopy**			*0.374*
Endometrial abnormalities at ultrasound	102 (36.6)	40 (41.7)	
Abnormal uterine bleeding	177 (63.4)	56 (58.3)	

HRT: hormonal replacement therapy. HSC-bio: hysteroscopically guided biopsy; HSC-res: hysteroscopic endometrial resection; OC: oral contraceptive
